# Dual Effects of ADHD Pharmacotherapy on Nutrition: A Scoping Review of Appetite, Eating Behavior, and Growth Across the Lifespan

**DOI:** 10.3390/nu18183039

**Published:** 2026-09-17

**Authors:** Jens-Mirco Engbrink, Janina Dapprich, Tobias Fischer, Anja Markant

**Affiliations:** Center for Nutrition and Therapy (NuT), University of Applied Sciences Muenster, Corrensstraße 25, 48149 Muenster, Germany; jm.engbrink@fh-muenster.de (J.-M.E.); janina.dapprich@fh-muenster.de (J.D.);

**Keywords:** ADHD, pharmacotherapy, dietary intake, eating behavior, nutritional status, growth, appetite

## Abstract

**Background**: Attention-deficit/hyperactivity disorder (ADHD) is associated with alterations in eating behavior, dietary patterns, and body weight regulation. Although pharmacological treatment is known to suppress appetite, its broader effects on nutrition-related outcomes remain unclear. This scoping review aimed to map the available evidence on dietary intake, eating behavior, nutritional status, growth, and bone health in individuals with ADHD receiving pharmacotherapy. **Methods**: A scoping review was conducted in accordance with the Joanna Briggs Institute methodology and PRISMA-ScR guidelines. PubMed and Scopus were searched from January 2010 to June 2026. Studies were eligible if they reported at least one nutrition-related outcome in medicated individuals with ADHD. Following study selection and data charting, the reported outcomes were grouped into four domains: dietary intake/eating behavior, nutritional status/metabolic biomarkers, growth/anthropometric outcomes, and bone health. **Results**: Twenty-seven studies met the inclusion criteria, the majority of which were observational and conducted in pediatric or mixed-age populations. Growth and anthropometric outcomes were the most frequently investigated domain. Stimulant treatment was associated with reduced appetite; lower energy intake during active treatment periods; and transient reductions in body weight, BMI, and growth velocity. Several studies also reported changes in eating behavior, including both increased risk of disordered eating and improvements in dietary structure. Evidence regarding nutritional biomarkers, metabolic outcomes, and bone health was limited and heterogeneous. **Conclusions**: ADHD pharmacotherapy is associated with a broad range of nutrition-related effects that extend beyond appetite suppression alone. The available evidence suggests that nutritional outcomes are shaped by a complex interaction of biological and behavioral mechanisms. Important evidence gaps remain regarding adult populations, long-term nutritional consequences, and nutritional biomarkers.

## 1. Introduction

Attention-deficit/hyperactivity disorder (ADHD) is a neurodevelopmental disorder characterized by persistent patterns of inattention, hyperactivity, and impulsivity that interfere with functioning across the lifespan [1]. Although ADHD is commonly diagnosed in childhood, symptoms frequently persist into adolescence and adulthood, contributing to impairments in educational, occupational, and social domains [2,3]. Beyond its cognitive and behavioral manifestations, ADHD has consistently been associated with alterations in eating behaviors and dietary patterns. Individuals with ADHD often exhibit irregular meal timing, impulsive eating, and a preference for energy-dense and highly palatable foods [4,5,6]. These patterns are thought to be linked to deficits in inhibitory control and reward processing, which may promote dysregulated eating independent of physiological hunger signals [7,8]. These behavioral characteristics may contribute not only to dysregulated eating patterns but also to adverse anthropometric outcomes. For example, ADHD symptom severity in early childhood has been associated with higher fat mass later in life [9].

Pharmacological treatment is a cornerstone of ADHD management, with stimulant medications such as methylphenidate (MPH) and amphetamines (AMP) demonstrating robust efficacy in reducing core symptoms [10,11]. However, these medications are also associated with side effects that directly affect appetite regulation and dietary intakes. Appetite suppression and reduced weight gain are among the most frequently reported effects, particularly in pediatric populations [12]. In addition to their effects on energy intake, pharmacotherapy-related changes in dietary behavior may have implications for the micronutrient status. Micronutrients such as iron and zinc play a critical role in dopaminergic neurotransmission and cognitive function, which are central to ADHD pathophysiology [13,14]. Alterations in micronutrient availability have been associated with ADHD symptom severity and treatment response in some studies, particularly in the context of iron deficiency or zinc status [15,16]. Despite the increasing research interest in nutrition and ADHD, the existing literature has predominantly focused on dietary interventions, elimination diets, and micronutrient supplementation as therapeutic strategies [17,18], with comparatively little systematic attention paid to pharmacotherapy itself as a determinant of dietary and nutrition outcomes. This raises a question that, to date, has rarely been addressed directly: if stimulant medications reduce appetite as a well-documented side effect, such pharmacotherapy could, in principle, also indirectly affect eating behavior, micronutrient status and/or growth. Although appetite suppression is a well-recognized adverse effect of stimulant treatment, the extent to which these changes influence downstream nutritional outcomes has not been systematically synthesized. Given the widespread and often long-term use of stimulant medication across the lifespan, understanding potential nutritional consequences is of considerable clinical relevance.

This gap may partly reflect differing disciplinary perspectives rather than a simple absence of data in the literature. Research on ADHD pharmacotherapy has predominantly originated from psychiatric and pharmacological traditions, which tend to frame appetite suppression as a known, clinically managed side effect of treatment rather than as a potential trigger for downstream nutritional consequences. Much of the existing literature on nutrition and ADHD, by contrast, has approached the relationship from the opposite causal direction, examining micronutrient or dietary factors as potential risk factors for ADHD, for example, maternal vitamin D status [19], prenatal dietary patterns [20], or prenatal folic supplementation as a preventive measure [21]. As a result, one literature documenting pharmacological appetite effects and another examining nutritional factors as antecedents of ADHD have developed largely in parallel, with limited direct integration. This scoping review addresses this issue explicitly from a nutritional science perspective, treating pharmacotherapy-induced appetite suppression not merely as a side effect to be managed but as a potential determinant of downstream nutritional outcomes warranting systematic investigation.

Therefore, a structured synthesis is needed to map the available evidence on nutrition-related outcomes reported in relation to pharmacological ADHD treatment across the lifespan. The overarching objective of this scoping review was to map the extent, range, and characteristics of the available evidence, including the types of nutrition-related outcomes investigated, their distribution across age groups, and areas in which evidence is limited or lacking.

## 2. Materials and Methods

### 2.1. Study Design

This scoping review followed the Joanna Briggs Institute (JBI) methodology for scoping reviews [22]. A broad inclusion strategy was applied to capture all available evidence on the nutritional aspects of individuals with ADHD receiving pharmacological treatment. The scoping review was also conducted and reported in accordance with the Preferred Reporting Items for Systematic Reviews and Meta-Analyses extension for Scoping Reviews (PRISMA-ScR) [23]. A formal protocol was developed for the review but was not prospectively registered or made publicly available. Primary research studies, systematic reviews and meta-analysis were considered during study selection. However, secondary publications were not included in the final data synthesis when the underlying primary studies were already represented to avoid double counting of the same data. Reviews and meta-analyses excluded because of overlapping primary evidence were used only for citation searching and contextual interpretation.

The review question was structured according to the Population–Concept–Context (PCC) framework. The population comprised individuals with ADHD across all age groups. The concept comprised nutrition-related outcomes reported in relation to pharmacological ADHD treatment, and the context encompassed all geographical, clinical, and research settings. The resulting review question was the following: What are the extent, range, and nature of the available evidence on nutrition-related outcomes reported in individuals with ADHD receiving pharmacological treatment across the lifespan?

### 2.2. Eligibility Criteria

Studies were eligible if they reported at least one nutrition-related outcome associated with ADHD pharmacological treatment. No individual outcome or outcome domain was specified as a mandatory inclusion criterion. Rather, specific nutrition-related outcomes were identified during data charting and subsequently grouped into conceptually related domains for evidence mapping.

Studies were included regardless of whether nutrition-related outcomes were assessed directly (e.g., dietary intake) or indirectly (e.g., anthropometric or metabolic outcomes). The chosen inclusion and exclusion criteria are presented in Table 1.

### 2.3. Information Sources and Search Strategies

To identify potentially relevant studies, systematic literature searches were conducted in the PubMed and Scopus databases. The search was restricted to studies published between January 2010 and June 2026 to reflect contemporary ADHD pharmacotherapy practices and ensure relevance to current clinical management. The search strategy was developed around three core concepts: ADHD, pharmacological treatment, and nutrition-related outcomes. The search strategy combined terms relating to ADHD, pharmacological treatment, and broad nutrition-related concepts, including diet, nutrition, dietary patterns, dietary intake, and eating. The initial search was conducted on 10 November 2025 and updated on 9 June 2026 to identify newly published studies. The complete search strategy is presented in Table A1 in Appendix A. All retrieved records were imported into the Rayyan Systematic Review Platform (Rayyan Systems Inc., Cambridge, MA, USA) for study management and duplicate removal. Titles and abstracts were screened according to the predefined eligibility criteria, followed by full-text assessment of potentially relevant studies. Following duplicate removal, title and abstract screening was conducted independently by two reviewers (J.M.E. and J.D.) according to the eligibility criteria.

### 2.4. Outcome Classification

To systematically map the evidence, nutrition-related outcomes were categorized into outcome domains. These domains were based on reported outcomes and aligned with the review objectives of assessing the effects of ADHD pharmacotherapy on nutrition-related parameters. The following outcome domains were defined:

1. Dietary intake and eating behavior (DE): This domain included studies that directly assessed dietary intake or eating patterns, such as food intake, nutrient intake, dietary patterns, meal frequency, or eating behavior, using dietary assessment instruments.
2. Nutritional status and metabolic biomarkers (NM): This domain included studies reporting nutrition-related biological or metabolic outcomes, such as micronutrient levels (e.g., iron and zinc), metabolic biomarkers (e.g., short-chain fatty acids), or indicators of nutritional status.
3. Growth and anthropometric outcomes (GA): Studies reporting anthropometric outcomes, including body weight, height, BMI, growth trajectories, and obesity prevalence, were classified as growth-related effects.
4. Bone health outcomes (BH): This domain included studies that assessed bone health outcomes, such as bone mineral density, bone mineral content, and other bone metabolism indicators.

### 2.5. Data Synthesis and Analysis

Data extraction was performed by the first author (J.M.E.) for all the included studies. A comprehensive Microsoft Excel data extraction form was developed prior to the extraction process. The form consisted of information on authors, the year of publication, country of origin where the study was conducted, study design, medication/intervention, sample size, population age, and key findings of the study. The extracted data were grouped into outcome domains and summarized within a scoping matrix. To facilitate the identification of research patterns and evidence gaps, the findings were visualized using a heatmap illustrating the distribution of studies across age groups, study design, and the outcome domains (DE, NM, GA, and BH). For comparison across studies, the participant populations were categorized into predefined age groups during data extraction. Studies were classified as including children (<11 years), adolescents (11–17 years), or adults (≥18 years) based on the reported age range or mean age of participants. This categorization was applied solely for the purpose of data synthesis, as age classifications varied across the included studies and no consistent framework was used across the literature. A critical appraisal of the included sources of evidence was not conducted, as the primary aim of this scoping review was to map the available evidence rather than to assess the methodological quality or risk of bias of individual studies, consistent with the objectives of the scoping review methodology.

## 3. Results

**Figure 1:**
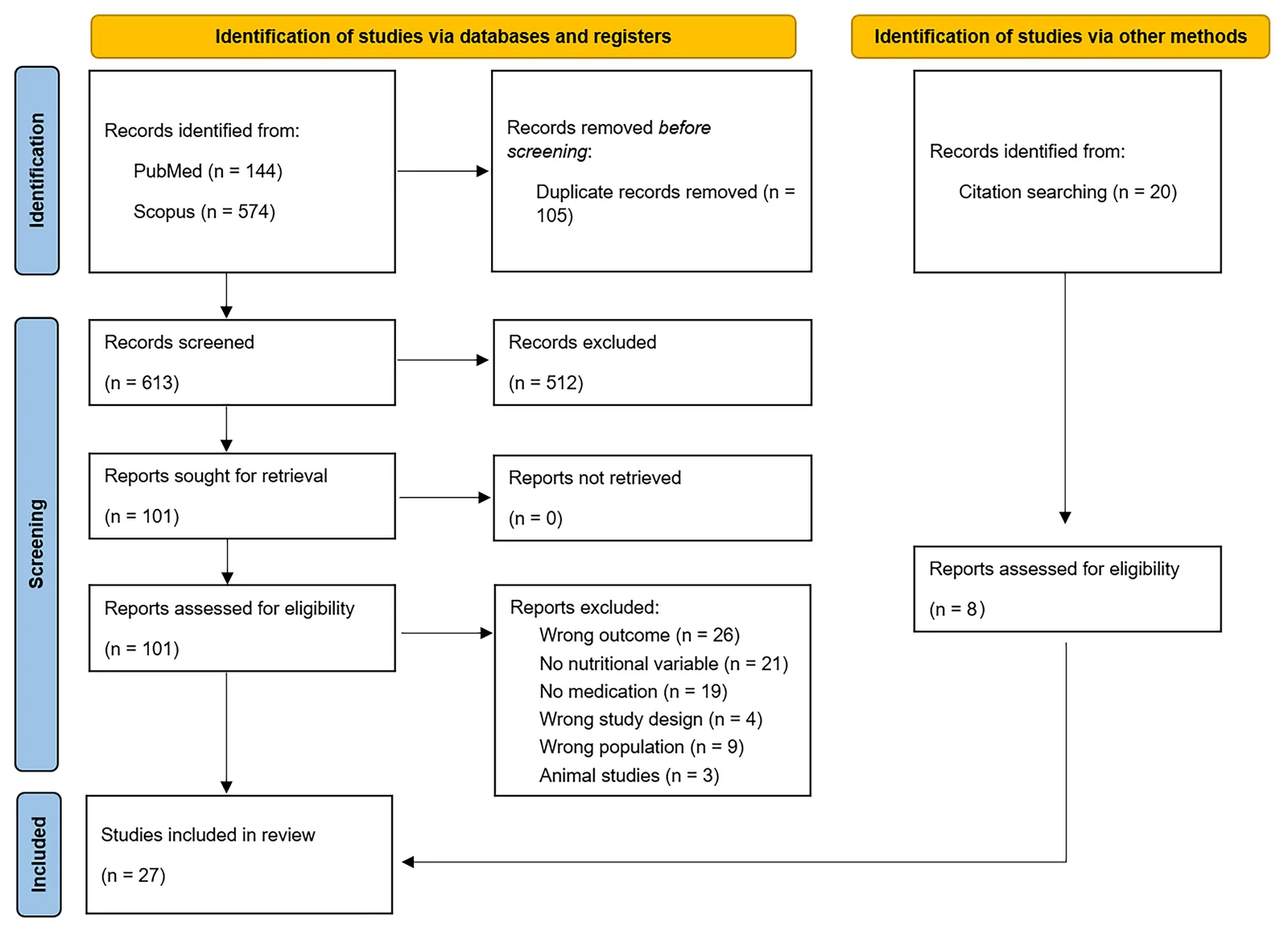
PRISMA flow diagram illustrating the study selection process, including the identification, screening, eligibility assessment, and final inclusion of studies in the scoping review [23].

The study selection process is presented in a PRISMA flow diagram (Figure 1). The database searches identified 718 records. Following the removal of 105 duplicates, 613 titles and abstracts were independently screened. A total of 101 records identified through the database searches underwent full-text assessment. The resulting eligibility decisions were reviewed, and 19 primary studies were included from the database searches. Citation searching identified 20 additional potentially eligible publications, all of which were assessed in full text. Twelve were excluded, and eight additional primary studies were included, resulting in a total of 27 included studies. All included studies were then imported into Citavi (Version 7.3.1). A summary of the study characteristics and key findings is presented in Table 2.

### 3.1. Evidence Distribution Across Outcome Domains

**Figure 2:**
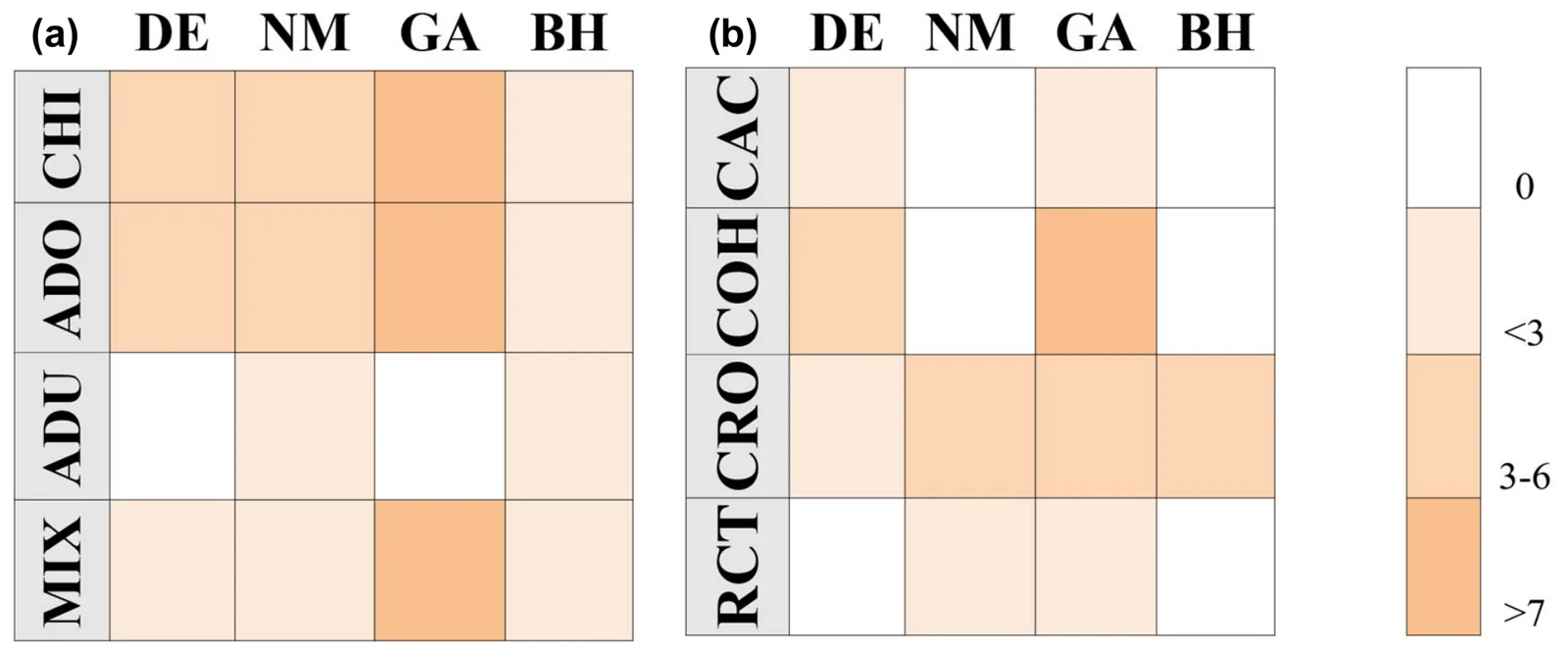
Heatmap of study distribution across outcome domains, (**a**) study design, and (**b**) population group. The color intensity represents the number of studies in each category. ADO, adolescents (11–17 y); ADU, adults (≥18 y); BH, bone health outcomes; CAC, case–control studies; CHI, children (<11 y); COH, cohort studies; CRO, cross-sectional studies; DE, dietary intake/eating behavior; GA, growth and anthropometric outcomes; MIX, mixed (all ages); NM, nutritional status and metabolic markers; RCT, randomized controlled trial.

The distribution of studies across outcome domains revealed substantial imbalances with respect to both population groups and study design (Figure 2). Most studies included mixed-age populations spanning childhood and adolescence, whereas only one study exclusively investigated children and two studies focused solely on adolescents. Adult populations were represented in only two studies, both of which examined nutritional/metabolic biomarkers or bone health outcomes [24,25]. Figure 2a further illustrates that mixed-age populations accounted for most studies across all outcome domains.

Most studies were observational in design (n = 25, 92.6%), predominantly comprising cohort studies (n = 15) and cross-sectional studies (n = 9). Only one case–control study and two randomized controlled trials were identified (Figure 2b).

**Figure 3:**
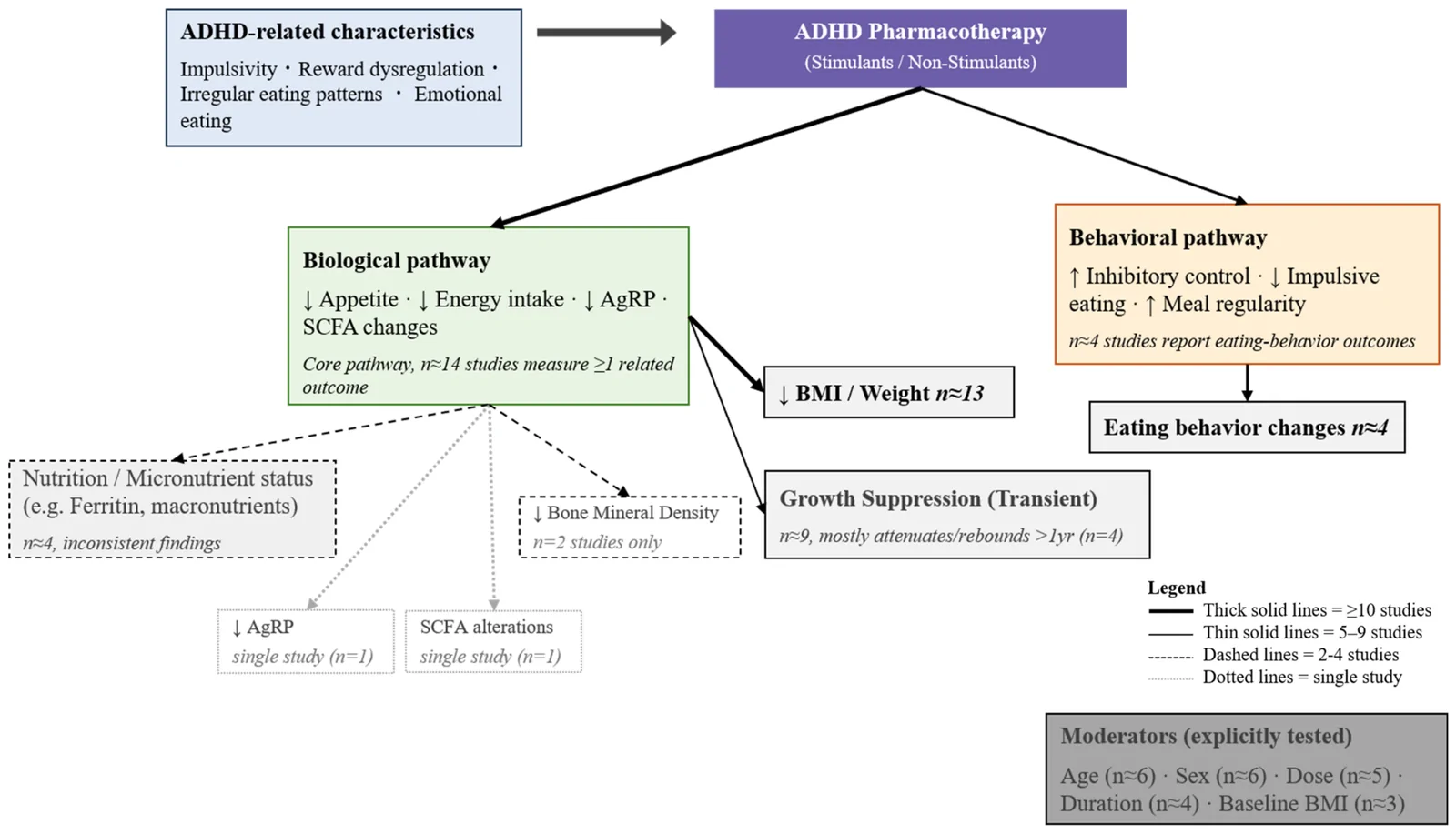
The evidence map illustrates the interaction between attention-deficit/hyperactivity disorder (ADHD) pharmacotherapy and nutrition-related outcomes synthesized from n = 27 included studies addressing biological and/or behavioral pathways. Numbers in parentheses indicate the number of studies reporting each outcome. Solid connectors indicate outcomes or relationships reported in three or more included studies, dashed connectors indicate those reported in two studies, and dotted connectors indicate those reported in one study. Line patterns reflect only the volume and distribution of the mapped literature and do not indicate evidence strength, certainty, or causality. All outcomes and relationships displayed in the figure were derived from the included primary studies; no external mechanistic evidence was incorporated. The grouping into biological and behavioral pathways represents a descriptive organization of the mapped findings rather than a causal model. Moderators are based on variables explicitly analyzed as effect modifiers in at least one included study. AgRP, agouti-related peptide; SCFA, short-chain fatty acids.

Growth and anthropometric outcomes represented the most frequently investigated domain (n = 19, 70.4%), followed by dietary intake/eating behavior (n = 5, 18.5%), nutritional status/metabolic biomarkers (n = 4, 14.8%), and bone health outcomes (n = 2, 7.4%). While growth-related outcomes were investigated across a broad range of study designs and population groups, dietary intake/eating behavior outcomes were predominantly examined in mixed-age populations. Bone health outcomes were identified only in observational studies and adult populations. Figure 3 provides a descriptive evidence map of the nutrition-related outcomes and relationships reported across the 27 included studies. The study counts indicate how frequently each outcome was represented in the mapped literature and should not be interpreted as an assessment of evidence strength or certainty.

### 3.2. Dietary Intake and Eating Behavior

Evidence on dietary intake is limited. Quantitative assessments of dietary intake are rare compared to studies examining eating behavior. Only one study assessed quantitative energy intake [26] and reported reduced caloric consumption during periods of active medication in adolescents with ADHD compared to non-medicated controls (1786.5 ± 335.9 vs. 2061.1 ± 242.4 kcal/day). Additionally, this study examined the temporal patterns of food intake, describing a compensatory increase in intake later in the day. Evening meals contributed a greater proportion of daily energy intake in medicated participants, accounting for approximately 15% more total daily energy intake than in unmedicated controls. In contrast, the majority of studies within DE (n = 4 of 5) focused on eating behavior, particularly disordered eating patterns [27,28,29,30]. These studies reported associations between pharmacological treatment and changes in eating-related symptoms, including an increased risk of disordered eating behavior [27,29], as well as improvements in dietary structure and regularity in others [27,29,31]. One study additionally examined nutrition-related behaviors and reported a higher prevalence of the use of multiple nutritional supplements among unmedicated individuals with ADHD compared with non-ADHD controls, whereas no increased supplement use was observed among medicated participants [32].

### 3.3. Nutritional Status and Metabolic Biomarkers

Evidence on nutritional status and metabolic biomarkers was limited and heterogeneous. Two studies reported alterations in metabolic and neuroendocrine biomarkers associated with ADHD pharmacotherapy. Gürbüzer et al. [31] observed significantly lower agouti-related peptide (AgRP) concentrations in MPH-treated individuals with ADHD compared with both untreated ADHD patients and healthy controls (*p* = 0.021). The study also reported higher low-density lipoprotein (LDL) concentrations in untreated ADHD participants than in medicated individuals (*p* < 0.001). In addition, Yang et al. [24] reported lower concentrations (extent was not reported) of short-chain fatty acids acetic acid (*p* = 0.010) and propionic acid (*p* = 0.0015) among children receiving stimulant treatment compared with children who had never received stimulants or had discontinued treatment. Findings regarding micronutrient-related biomarkers were inconsistent. One study reported no significant differences in ferritin (*p* = 0.72) or transferrin concentrations (*p* = 0.09) between groups [25]. In contrast, Rosenau et al. [33] observed a decline in ferritin concentrations following withdrawal of MPH treatment after seven weeks (−17.5 μg/L; *p* = 0.0057). No consistent pattern was observed across studies investigating micronutrient-related biomarkers.

### 3.4. Growth and Anthropometric Outcomes

Growth and anthropometric outcomes were the most frequently investigated outcome domain (n = 19, 70.4%). Several studies reported reductions in BMI z-scores in children ranging from −0.39 to −0.58 and decreases in annualized height velocity ranging from −0.43 to −0.86 cm/year following initiation of stimulant treatment [26,30,34,35,36,37,38,39,40,41,42,43,44]. These changes were generally most pronounced within the first 6–12 months of treatment.

The magnitude of these effects appeared to vary across subgroups. Greater reductions in BMI and weight were reported among younger children [40,44] and individuals with higher baseline body weight [29,36,37,39,43]. Findings regarding sex differences were mixed. Two studies reported greater reductions in height, weight, or BMI among females [35,38,40], whereas four studies observed no significant sex-related differences [29,34,44,45]. In addition, amphetamine-based medications were associated with greater early weight loss than methylphenidate or atomoxetine [30,34].

Longitudinal studies suggested attenuation of growth-related effects over time, with partial or complete catch-up growth, particularly for body weight [36,40]. Dura-Travé et al. [46] similarly described growth suppression as a transient and reversible effect, with evidence of rebound growth after approximately 36 months of treatment. Kousha et al. [47] reported an increase in BMI of 4.69 kg/m^2^ after six years of treatment (*p* = 0.008). Zhang et al. reported an inverse association between treatment durations and height gain, with each additional year of MPH treatment associated with an estimated 0.344 cm greater height difference [30,44]. Granato et al. [37] additionally reported lower rates of overweight and obesity among stimulant-treated individuals and more favorable BMI trajectories in participants with elevated baseline weight. McCarthy et al. [48] found that boys aged 6–10 years receiving MPH for <12 months had higher odds of BMI ≤ 3rd percentile than non-ADHD controls (OR, 4.52; 95% CI, 1.54–13.28; *p* = 0.040).

### 3.5. Bone Health Outcomes

Bone health outcomes were examined in only two studies (n = 2, 7.4%). Howard et al. [49] reported lower bone mineral density (BMD) and a higher prevalence of osteopenia among medicated children compared with non-medicated controls (38.4% vs. 21.6%, *p* < 0.01). In adults, Lawson et al. [50] observed modest reductions in BMD in axial skeletal regions, including the skull and thoracic spine (approximately 6%), whereas no consistent differences were identified in appendicular skeletal sites. No study evaluated longitudinal changes in bone health outcomes following initiation or discontinuation of ADHD pharmacotherapy.

## 4. Discussion

A key finding of this scoping review is the dual and partly opposing nature of pharmacological treatment effect in individuals with ADHD. While stimulant medication is consistently associated with reduced caloric intake during periods of active treatment [26], it may simultaneously improve behavioral regulation and executive functioning, potentially promoting more structured eating behaviors and reducing impulsive food consumption. Consequently, several studies have reported lower rates of overweight and obesity among medicated individuals with ADHD compared with their non-medicated counterparts [51,52]. This apparent paradox may be explained by both behavioral and neurobiological mechanisms. From a behavioral perspective, improved inhibitory control and reduced impulsivity may facilitate healthier dietary choices and more regular meal patterns. From a neurobiological perspective, ADHD has been linked to alteration in dopaminergic reward pathways, which may contribute to reward-seeking eating behaviors and overeating. The appetite-suppressing properties of stimulant medications are further illustrated by their misuse for weight-control purposes. Previous research has shown that approximately 12% of adults report non-medical use of prescription ADHD medication for weight loss [53], while nearly one in six female college students aged 16–24 years reported amphetamine use for weight-control purposes [54]. Although these findings originate from non-clinical populations, they underscore the well-recognized anorexigenic effects of stimulant compounds.

Differences in pharmacokinetic profiles may further contribute to the heterogeneity of nutrition-related outcomes observed across studies. Immediate-release (IR) methylphenidate produces a rapid increase in plasma concentration and dopamine transporter blockade, whereas extended-release (ER) and osmotic-release oral system (OROS) formulations provide a more gradual and sustained exposure [55,56,57,58]. These differences may influence both the magnitude and duration of appetite suppression. In particular, prolonged appetite suppression during the day may contribute to compensatory caloric intake during the evening as medication effects decline [26]. Similar considerations apply to LDX, a long-acting prodrug that is converted to d-amphetamine in the bloodstream and provides a more stable pharmacological effect throughout the day [59]. Beyond appetite suppression, LDX has been shown to enhance satiety, reduce food-related reward responsiveness, and improve cognitive control, mechanisms that may contribute to its established efficacy in binge-eating disorder and reflected by international guidelines recommending LDX as a pharmacological treatment option for BED [60,61]. Supporting these observations, a recent network meta-analysis found that amphetamine-based medications were associated with a greater risk of weight loss than methylphenidate formulations [62]. Collectively, these findings suggest that nutritional outcomes may vary not only according to medication use per se but also according to the specific pharmacological properties of individual stimulant formulations.

Beyond direct pharmacological effects on appetite, improvements in ADHD symptoms may indirectly influence eating behavior and dietary patterns. Individuals with ADHD frequently exhibit impulsive decision-making, heightened reward sensitivity, and difficulties delaying gratification, factors that may contribute to unhealthy eating behaviors and excessive consumption of highly palatable foods [63]. In addition, ADHD is commonly associated with emotional dysregulation and psychiatric comorbidities such as depression, both of which have been linked to emotional eating and dysregulated food intake [64]. Consequently, improvements in executive functioning and self-regulation during pharmacological treatment may extend beyond symptom control and facilitate healthier eating behaviors. Furthermore, highly palatable foods may represent an alternative source of reward and reinforcement in some individuals with ADHD, potentially contributing to overeating when symptoms remain insufficiently controlled. Sex-specific biological factors may also contribute to variability in nutrition-related outcomes. Female sex hormones have been shown to modulate dopaminergic and serotonergic signaling pathways involved in responses to psychostimulant treatment [51], suggesting that treatment effects on appetite and eating behavior may differ between males and females. Together, these findings indicate that nutrition-related outcomes in ADHD are shaped not only by medication-induced changes in appetite but also by broader behavioral and psychosocial mechanisms that influence food-related decision-making. External evidence from anorexia nervosa suggests that reduced adipose tissue mass and the resulting hyperleptinemia may be associated with increased physical activity and an increased drive for activity, although the strength and direction of this relationship in humans remain incompletely understood [65]. Preliminary off-label observations further suggest that Metreleptin administration may reduce starvation-related behavioral symptoms, including the drive for activity, but controlled clinical trials are required before therapeutic conclusions can be drawn. These findings illustrate that apparent hyperactivity may, in some contexts, reflect metabolic adaptations to energy deficiency rather than exclusively a primary neurobehavioral phenotype.

As previously noted, reduced brain dopaminergic activity plays a central role in ADHD pathophysiology, predisposing these individuals to reward deficiency syndrome. Therefore, overeating might be a form of self-medication for patients with ADHD to compensate, at least temporarily, for dopamine deficits. Several appetite-regulating peptides may contribute to medication-related changes in food intake and body weight. Among these, AgRP is an orexigenic neuropeptide involved in the regulation of appetite and energy balance [66,67,68]. Findings from the included studies suggest that stimulant treatment may influence pathways related to appetite regulation, a notion further supported by alterations in leptin and ghrelin concentrations reported by Gurbuz et al. [69]. After three months of treatment, leptin concentrations increased, whereas ghrelin concentrations decreased, accompanied by appetite suppression in approximately 70% of medicated children with ADHD. These changes were associated with reductions in BMI, body weight, and circulating Insulin-like growth factor 1 (IGF-1) concentrations. Similarly, Sahin et al. [70] proposed that alterations in ghrelin and adiponectin signaling may contribute to methylphenidate-associated appetite suppression and weight loss.

Another noteworthy finding of this scoping review was the emerging evidence that ADHD pharmacotherapy may influence metabolic processes beyond appetite regulation and energy intake. Included studies reported alterations in short-chain fatty acid (SCFA) concentrations following stimulant treatment, suggesting a potential involvement of the microbiota–gut–brain axis in treatment-related nutritional outcomes [71,72]. Although only a small number of studies investigated these parameters, the findings indicate that pharmacotherapy may affect biological pathways extending beyond the established dopaminergic and appetite-regulating mechanisms discussed above. This observation is consistent with emerging evidence highlighting the role of the microbiota–gut–brain axis in neurodevelopmental and psychiatric disorders, where microbial metabolites have been implicated in metabolic regulation, immune function, and neurobiological signaling. However, the clinical significance of these findings remains unclear, and the available evidence is currently insufficient to determine whether changes in SCFA profiles contribute directly to alterations in nutritional status, eating behavior, or long-term metabolic health in individuals with ADHD. Therefore, further studies are required to clarify the relevance of microbiota-related mechanisms and their potential role in mediating treatment-associated nutritional outcomes. The observed ferritin findings may be particularly relevant in the context of ADHD pathophysiology, given the reported association between iron status, dopamine transporter availability, and dopaminergic signaling [26,33], all of which are closely related to the therapeutic mechanism of MPH. Following randomized withdrawal of long-term MPH-treatment, the ferritin concentration decreased, while higher baseline ferritin and zinc concentrations moderated changes in behavioral symptoms and working-memory performance, respectively. From a mechanistic perspective, iron acts as a cofactor for tyrosine hydroxylase, the rate-limiting enzyme in catecholamine synthesis that catalyzes the conversion of tyrosine to L-DOPA, a precursor of dopamine [73]. This provides a possible biological context for an interaction between iron status and dopaminergic treatment pathways. However, the study demonstrates that the observed change in serum ferritin, altered central dopamine synthesis, and serum ferritin is not a direct measure of brain iron availability. Moreover, participants did not exhibit iron or zinc deficiency. Therefore, these preliminary findings should not be interpreted as evidence supporting routine supplementation during MPH treatment or withdrawal.

Although growth and anthropometric outcomes constitute the most frequently investigated domain, the clinical interpretations of these findings remain less straightforward than the volume of research might suggest. The observation that changes were generally most pronounced during the early stages of treatment and attenuated in several longitudinal studies may indicate an initial treatment-associated perturbation rather than a uniform and permanently altered growth trajectory. This interpretation is consistent with an external systematic review and meta-analysis by Carucci et al. [74], which identified statistically detectable reductions in height and weight during long-term methylphenidate treatment but concluded that the effects sizes were small and potentially of limited clinical relevance. The tendency for changes in body weight to precede or exceed changes in height supports reduced energy intake as one plausible contributing pathway. However, this temporal pattern does not establish causality, as baseline BMI, age, pubertal development, ADHD symptom severity, medication dose, treatment interruptions, and other clinical adaptations may also influence individual growth trajectories. Poulton et al. [75] similarly reported slower growth during stimulant treatment without a corresponding delay in bone maturation, suggesting that reduced growth velocity should not automatically be interpreted as generalized developmental delay. Moreover, a lower BMI or a lower prevalence of being overweight among medicated individuals cannot be classified as uniformly beneficial or adverse without considering baseline nutritional status. The same anthropometric change may represent an improvement in a participant with obesity but a clinically relevant concern in a participant with normal or low baseline weight.

Unlike the relatively consistent findings regarding appetite suppression and weight-related outcomes, evidence concerning bone health was more limited and yielded less consistent results. Nevertheless, the available studies suggest that stimulant treatment may influence skeletal health through mechanisms extending beyond reduced energy intake alone. One proposed explanation is a lower intake of nutrients relevant to bone metabolism, particularly calcium and vitamin D, secondary to medication-induced appetite suppression. However, this interpretation should be considered with caution, as vitamin D insufficiency is highly prevalent in the general population and may therefore represent a background risk factor rather than a medication-specific effect [76]. However, alterations in sympathetic nervous system activity may represent an additional biological pathway. The sympathetic nervous system plays an important role in bone remodeling by regulating the balance between osteoblast-mediated bone formation and osteoclast-mediated bone resorption [77,78,79]. Increased sympathetic activity has been associated with enhanced bone resorption and reduced bone formation, potentially contributing to reductions in bone mineral density. Although some studies reported modest reductions in BMD, findings were inconsistent and appeared to vary according to treatment duration. For example, Feuer et al. [80] observed that BMD deficits were no longer evident when stimulant exposure was shorter than three months. Given the small number of available studies and the heterogeneity of outcome measures, robust conclusions regarding the long-term effects of ADHD pharmacotherapy on bone health cannot yet be drawn. The evidence map (Figure 3) synthesizes the available evidence into two interacting pathways through which ADHD pharmacotherapy may influence nutrition-related outcomes. While the biological pathway is characterized primarily by appetite suppression, reduced energy intake, and transient growth effects, the behavioral pathway reflects changes in meal regularity, impulsive eating, and dietary regulation. Together, these pathways provide a potential explanation for the heterogeneity observed across studies. This may explain the heterogeneity observed across studies and highlights the importance of considering multiple pathways simultaneously when evaluating nutrition-related outcomes of ADHD pharmacotherapy.

These findings also have practical implications for nutrition counseling and clinical care. In children and adolescents, weight, height, BMI, and growth velocity should be monitored throughout pharmacological treatment, particularly during treatment initiation and dose adjustment. In adults, monitoring should focus primarily on changes in body weight, BMI, appetite, and dietary intake. Assessment of appetite should consider its variation across the day, as reduced intake during periods of active medication may be followed by increased evening intake when the effects of medication decline. Patients and families may benefit from practical nutritional education focused on maintaining dietary quality and providing nutrient- and energy-dense food during periods of greater appetite. Persistent weight loss, growth deceleration or markedly restricted dietary intake may warrant individualized nutritional assessment. Micronutrient supplementation should not be initiated solely based on stimulant treatment but should be guided by dietary assessment; clinical indications; and, where appropriate, biochemical evaluation.

This review has several strengths and limitations that should be considered when interpreting the findings. A strength is the structured mapping of nutrition-related outcomes across multiple domains and age groups. This approach enabled the identification of areas in which research is concentrated and of outcomes and populations for which evidence remains limited. There is a strong imbalance in the studied populations, with most studies focusing on children, while evidence in adults is limited. This is particularly important considering the increasing recognition of ADHD as a condition that persists across the lifespan. Moreover, the research has predominantly focused on growth and anthropometric outcomes, whereas comparatively fewer studies have directly examined dietary intake and eating behavior, and only a small number have investigated nutritional biomarkers or bone health. Additionally, the evidence base is largely dominated by observational study designs, with relatively few randomized controlled trials. This limits the ability to draw causal inferences regarding the effects of pharmacotherapy on nutrition-related results. The substantial heterogeneity in outcome definitions and measurement methods, particularly in the assessment of dietary intake and eating behavior, hampers comparability between studies. Comparisons of findings across age groups should be interpreted with caution, as the included studies used heterogenous age ranges and definitions of participant populations. To improve comparability, age categories were harmonized during data synthesis; however, some degree of misclassification cannot be excluded from this study.

No finalized protocol was prospectively registered or published, which limits the ability to independently assess potential deviations from originally planned review procedures. The included studies varied considerably with respect to participant characteristics, age ranges, medication types, treatment duration, and outcome measures, which limited direct comparability across studies. Although age categories were harmonized during data synthesis to facilitate interpretation, some degree of misclassification cannot be excluded. The database search was limited to PubMed and Scopus. Although these databases provide complementary biomedical and multidisciplinary coverage and the search was supplemented by citation searching, potentially relevant studies indexed exclusively in other databases, such as PsycINFO, CINAHL, or Web of Science, may have been missed. Because the database search relied on broad diet- and nutrition-related terminology, studies indexed exclusively under more specific outcome terms may not have been retrieved. Citation searching was used to identify additional relevant primary studies; nevertheless, incomplete retrieval cannot be excluded.

## 5. Conclusions

ADHD pharmacotherapy is associated with a broad range of nutrition-related outcomes that extend beyond appetite suppression alone. The available evidence indicates that dietary intake, eating behavior, growth trajectories, and metabolic processes may be influenced by a complex interaction of pharmacological, biological, and behavioral mechanisms. While short-term reductions in energy intake, body weight, and growth velocity were consistently reported, these effects frequently attenuated over time and were often followed by partial catch-up growth. Current evidence regarding nutritional biomarkers, bone health, and long-term nutritional consequences remains limited and heterogeneous, particularly in adult populations. The findings of this review highlight the need for a more integrated research approach that bridges nutritional and psychiatric perspectives and incorporates behavioral, biological, and nutritional outcomes simultaneously. Such an approach may improve our understanding of how ADHD pharmacotherapy shapes nutritional health across the lifespan and help inform future monitoring and intervention strategies.

## Figures and Tables

**Table 1:** Inclusion and exclusion criteria of studies.

Inclusion Criteria	Exclusion Criteria
Individuals diagnosed with ADHD	Studies not including individuals diagnosed with ADHD
Studies involving ADHD pharmacotherapy (e.g., methylphenidate, amphetamines, atomoxetine, guanfacine, or clonidine)	Studies including only non-medicated ADHD populations without assessing medication exposure
At least one nutrition-related outcome, including appetite, eating behavior, dietary intake, nutritional status, micronutrient or metabolic biomarkers, anthropometric measures (e.g., weight, height, BMI), growth, or bone health	Studies not reporting any nutrition-related outcome (e.g., no anthropometric, metabolic, nutritional, or dietary variables) Systematic reviews and meta-analyses whose underlying primary studies were already represented in the evidence synthesis
Original primary research, as well as systematic reviews and meta-analyses for supplementary evidence identification	Commentaries, editorials, letters, conference abstracts without sufficient data, narrative reviews without systematic synthesis, or case reports with fewer than ten participants
Studies conducted in human populations	Animal or in vitro studies
Articles published in peer-reviewed journals	Articles not published in peer-reviewed journals
Articles published in English	Articles not published in English

ADHD, Attention-deficit/hyperactivity disorder; BMI, body mass index.

**Table 2:** Summary of study characteristics and key findings related to dietary intake, eating behavior, nutritional status, metabolic biomarkers, growth and anthropometric outcomes, and bone health among individuals with ADHD receiving pharmacological treatment.

Author, Year	Country, Study Design	Population, Age Range	Medication/Intervention	Key Result
[24] Yang et al., 2022	SE, CRO	88 children with ADHD, 145 adults with ADHD; 36 healthy adult CG 5–55 y	None (observational); factors analyzed: antibiotics (past 2 y), diet (PC1–3), vitamin D, delivery mode, and ADHD MED.	Adults with ADHD showed lower plasma SCFAs (formic/acetic/propionic/succinic); antibiotics, age, and stimulants suppress SCFAs; dietary fiber boosts acetic acid; C-section was associated with lower succinic acid levels; vitamin D linked to formic acid.
[25] Menegassi et al., 2010	BR, CRO	19 children/adolescents on MPH (3 months), 22 drug-naïve ADHD, 21 healthy CG 6–15 y	MPH-IR; dose unspecified	No evidence of iron deficiency or dietary iron deficits in ADHD was reported; RDW elevation in MPH-naïve ADHD (not clinically significant); no MPH-induced iron depletion after 3 months; no link between ADHD severity and ferritin.
[26] Durá-Travé et al., 2014	ES, CAC	100 ADHD patients (DSM-IV-R) on MPH-ER (68 boys; 32 girls) 100 healthy CG 11–12 y	MPH-ER: MPH-OROS (63%; 1.07 mg/kg/day) or MPH-MR (37%; 0.90 mg/kg/day); no washout.	Compared with controls, children receiving ER-MPH had a lower total energy intake (1786.5 vs. 2061.1 kcal/day; difference of −274.6 kcal/d). Energy intake was lower early in the day but higher at supper (+99.4 kcal).
[27] Alsindi et al., 2024	BH, LT	61 adolescents (86.9% male) with ADHD (DSM-IV + Conner’s test) 10–19 y	MPH: 41% at T1 → 23% at T2 (all male users at T2); EAT-26: 26-item screening tool (score > 20 = at risk).	Risk of disordered eating increased by 23% during follow-up and showed a greater increase among males. MPH use decreased significantly >3 y (41% vs. 23%; *p* < 0.001), while BMI category (healthy/unhealthy) was not significantly associated with risk of disordered eating.
[28] Bisset et al., 2019	AU, LT	230 ADHD vs. 2742 healthy CG 8–13 y	ADHD MED exposure [19.6% at 8–9 y (13% MPH; 3% AMP) → 24.8% at 10–11 y (18.7 MPH; 4.3% AMP; 1.7% ATX) → 30% at 12–13 y (25.2 MPH; 3.9% AMP; 0.9% ATX)]. Pictorial Body Image Instrument	Children with ADHD had higher odds of body dissatisfaction (perceived/desired body image score range of 1–7) than CG at ages 8–9 y (OR, 1.5; *p* = 0.05) and 12–13 y (OR, 1.7; *p* = 0.006), independent of BMI and comorbidities. Medicated children had a 2.8-fold higher adjusted odds of trying to gain weight.
[29] Aykutlu et al., 2024	TR, CRO	547 ADHD children: 152 drug-naive, 156 STG, 53 SATG, 186 CG 6–12 y	STG: MPH 20 mg/day (median), 12 months; SATG: MPH 27 mg/day + risperidone 1 mg/day (85%) or aripiprazole 4 mg/day (15%), 15 months.	ADHD increases obesity risk (13.8% in drug-naïve group vs. 8.6% in CG); the stimulant group had a lower prevalence of obesity (4.5% in STG); antipsychotics attenuate this effect (7.5% in SATG); disordered eating persists in SATG (high EO/DD); and stimulants normalize eating behaviors (STG = CG).
[30] Deng et al., 2021	CN, LT	86 children/adolescents with ADHD treated with MPH (n = 57) or ATX (n = 29) for ≥24 weeks 6–18 y	MPH: Concerta (OROS; 18–54 mg/day); ATX: Strattera (0.5–1.4 mg/kg/day)	At six months, decreases in weight and BMI z-scores were greater with MPH (−0.39 and 0.43) than with ATX (0.07 and 0.08). Height changes were comparable between treatments, while later weight and BMI rebound was observed in the ATX group.
[31] Gürbüzer et al., 2023	TR, CRO	117 adults (70 ADHD: 32 treated with MPH, 38 untreated; 47 healthy CG)18–65 y	≥6 months (treated group); 1-day washout pre-blood draw	MPH-treated adults with ADHD had lower AgRP concentrations than untreated participants with ADHD and healthy controls (*p* = 0.021). Untreated participants with ADHD had higher LDL cholesterol concentrations than medicated participants (*p* < 0.001). Disordered eating and night-eating syndrome were reported in 8.6% and 26.9% of participants with ADHD, respectively.
[32] Scholle et al., 2019	DE, CRO	51 medicated ADHD vs. 76 unmedicated ADHD children/adolescents 5–17 y	MPH; ATX; AMP (amphetamine, dexamphetamine, lisdexamphetamine)	Unmedicated participants with ADHD had a 2.6-fold higher likelihood of using multiple nutritional supplements than participants without ADHD; no multiple NUS use was reported in medicated ADHD.
[33] Rosenau et al., 2022	NL, RCT	63 children/adolescents with ADHD on MPH ≥ 2 years: 33 randomized to gradual withdrawal (placebo), 30 to continued methylphenidate 8–18 y	63 children/adolescents with ADHD on MPH ≥ 2 years: 33 randomized to gradual withdrawal (placebo), 30 to continued methylphenidate 8–18 y	MPH withdrawal reduces ferritin (−17.5 μg/L); high baseline ferritin/zinc predicts worse symptoms/cognition after withdrawal; ferritin/zinc may be biomarkers for MPH dependence; no iron/zinc deficiency in this cohort.
[34] Al Eid et al., 2024	AE, LT	78 children/adolescents with ADHD on stimulants (MPH-IR/-OROS, AMP-IR/-ER) and 8 ADHD CG 6–18 y	MPH-IR (n = 37) MPH-OROS (n = 27) AMP-IR (n = 3) AMP-ER (n = 11)	Initial weight loss was observed after 4 months of stimulant treatment followed by gradual recovery and stabilization of weight-for-age z-scores by 12 months. Early weight loss was greater with amphetamine-based medications than with MPH. Age and time but not sex, autism spectrum disorder, or sleep problems, were significant predictors of weight change.
[35] Díez-Suárez et al., 2017	ES, LT	342 children/adolescents with ADHD treated with MPH for 27 months (IQR: 14–41); no CG 6–18 y	MPH: 1.25 ± 0.40 mg/kg/day (total: 59.7 ± 22.9 mg/day); mixed MPH formulation.	Weight and BMI decreased by approximately 0.4–0.6 SDS during MPH treatment. Height SDS reductions were observed among children aged < 12 y and among females. Greater changes were associated with higher doses (1.25+ mg/kg/day) and longer treatment. The overall change in height SDS was small (−0.064).
[36] Faraone et al., 2010	US, LT	281 children with ADHD treated with LDX for up to 15 months; no CG 6–13 y	LDX: (30, 50, or 70 mg/day; titrated) BMI/height z-scores (CDC)	During 15 months of LDX treatment, weight z-scores decreased from 0.55 to 0.07, and BMI z-scores decreased from 0.62 to −0.03. Height gain was 3.9 cm, compared with an expected 4.8 cm. Changes were greatest during the first six months and attenuated after 12 months, tallest/heaviest children were most affected, prior stimulant exposure was protective, and no severe stunting was observed.
[37] Granato et al., 2018	BR, RS	93 children/adolescents with ADHD vs. 334 CG; BMI/height z-scores (WHO AnthroPlus) 5–14 y	MPH: Not specified (likely IR); dose not reported; 2.6 ± 1.5 years duration.	Overweight or obesity was more prevalent among participants with ADHD than controls (40.9% vs. 34.7%, *p* < 0.05). MPH-treated participants showed a mean BMI z-score reduction of 0.390 (*p* < 0.01) without a significant height change. Among medicated participants with baseline overweight or obesity, 42.1% moved to a lower BMI category.
[38] Kim et al., 2014	KR, LT	157 Korean children/adolescents with ADHD treated with MPH for 12–88 months; no CG 5–14 y	MPH: 0.98 ± 0.27 mg/kg/day (OROS/ER/IR formulations).	MPH was associated with modest, first-year-dominated suppression (weight: −0.109 z-score/y; height: −0.072 z-score/y); girls were more vulnerable to weight loss; no dose/formulation differences were observed; depressive disorder was protective for height; and no permanent stunting was observed.
[39] Landgren et al., 2017	SE, LT	70 children/adolescents (87.1% male) with ADHD treated with MPH for 3.3 y; no CG 8–18 y	MPH-ER/-OROS Dose: 0.95 ± 0.4 mg/kg/day (range: 0.35–2.6)	Mean height z-score decreased by 0.3, and BMI z-score decreased from 0.8 to 0.3. Among initially overweight participants, 50% reached a normal BMI category, whereas 9% of the total sample developed underweight.
[40] Lee et al., 2022	KR, LT	82 drug-naive Korean children/adolescents with ADHD treated with MPH for 24 months; no CG 6–15 y	MPH-ER/-OROS/-IR; dose not reported	Over 24 months, MPH treatment was associated with decreases in mean height and weight z-scores of 0.186 and 0.405, respectively (*p* = 0.001). Larger changes were observed among younger children and females, whereas partial rebound was reported among adolescents.
[41] Lentferink et al., 2018	NL, RS	298 children/adolescents with ADHD; no CG 4–18 y	MPH: 79% IR, 21% SR; 0.5 mg/kg/day (range: 0.2–1.4); max 2.0 mg/kg/day; 18-month follow-up.	During MPH treatment, BMI SDS and height SDS decreased by 0.4 and 0.2, respectively. BMI SDS remained stable among participants who were underweight at baseline, and only a weak dose–response association was observed.
[42] Waxmonsky et al., 2020	US RCT	230 children with ADHD (DSM-IV): 180 randomized to MED (OROS-MPH), 50 to BT 5–12 y	OROS-MPH (primary; 0.97 mg/kg/day); WRT: MON (monthly checks), DH (school-day-only MED), and CS (150-kcal evening supplement + MED).	Before initiation of the weight recovery interventions, stimulant treatment was associated with reductions in standardized weight and height. Drug holidays and caloric supplementation increased weight velocity, but none of the weight-recovery interventions significantly increased height velocity.
[43] Mellström et al., 2020	SE, RS	724 children (<18 y) with ADHD vs. 8508 reference children 4–18 y	MPH: 60% IR, 40% ER; 0.81 mg/kg/day; up to 3 years; individually titrated.	ADHD was associated with higher odds of overweight or obesity (OR, 1.87). MPH treatment was associated with reductions in BMI SDS of 0.51 overall and 0.64 among participants with baseline overweight or obesity. Larger reductions were associated with higher baseline BMI-SDS.
[44] Zhang et al., 2010	CN, LT	146 Chinese children with ADHD treated with MPH for 2–4 y vs. 29 drug-free ADHD CG 6–12 y (pre-pubertal; Tanner Stage I)	MPH-IR: 10–20 mg/day (0.27–0.64 mg/kg/day Non-drug therapies (n = 29)	MPH was associated with dose-dependent growth suppression: a height deficit of −1.86 cm over 2–4 y, with the greatest suppression in Year 1 (4.28 cm/y). Weight suppression (−2.16 kg) was not mediated by malnutrition (weight for height unchanged) Longer treatment → greater deficits (regression: 0.344 cm/y)
[45] Kang et al., 2016	KR, CRO	50 children with ADHD on MPH (K-ARS), 69 drug-naïve ADHD, 60 healthy CG 7–12 y	MPH dose: 0.91 ± 0.37 mg/kg/day; duration: 14.2 ± 1.6 months; drug-naïve ADHD: no prior MPH; CG: age/sex-matched.	No significant differences in height, weight, or BMI were observed between MPH-treated children, drug-naïve children with ADHD, and healthy controls.
[46] Durá-Travé et al., 2012	ES, LT	187 children with ADHD; weight/height deficits vs. Spanish norms (Centro Andrea Prader) 6–10 y	OROS-MPH: 1.31 ± 0.2 mg/kg/day; once daily; 48-month follow-up; no drug holidays.	OROS-MPH was associated with transient, reversible growth suppression (max deficits at 30 months: 4.274 kg weight, 2.69 cm height); rebound after 36 months.
[47] Kousha et al., 2024	IN, RS	149 children/adolescents with ADHD (DSM-IV-TR); no CG 3–18 y	MPH: Not specified; dose not reported; 1–6 years duration.	BMI increased by 4.69 kg/m^2^ over six years of MPH treatment (*p* = 0.008), with greater increases associated with longer treatment duration. No significant differences according to sex or ADHD subtype were observed.
[48] McCarthy et al., 2018	DE, CRO	4244 boys: 65 children with ADHD on MPH (<12 mo) vs. 53 children (≥12 mo) vs. 320 ADHD CG vs. 3806 non-ADHD CG 6–15 y	MPH < 12 months (short-term) vs. ≥12 months (long-term); dosage/formulation unspecifiedCG: ADHD without current MPH; non-ADHD	Compared with controls, boys aged 6–10 years receiving MPH > 12 months had higher odds of BMI ≤ 3rd percentile (OR, 4.52; 95% CI, 1.54–13.28; *p* = 0.006). Boys aged 11–15 years receiving MPH > 12 months also had higher odds (OR, 3.59; 95% CI, 1.06–12.22; *p* = 0.040). No significant associations with short stature were observed.
[49] Howard et al., 2017	US, CRO	5315 children/adolescents; propensity-matched subsample (n = 1967); 167 on ADHD MED (stimulants/atomoxetine); 4171 non-MED; 977 on other MED. 8–17 y	MPH, MPX, and AMP (dextroamphetamine, atomoxetine, and lisdexamfetamine); self-reported use in past month	ADHD medication use is linked to ~0.5 SD lower BMD (femur/neck), and osteopenia was observed in 38.4% of participants (vs. 21.6% non-MED).
[50] Lawson et al., 2022	US, CRO	7961 adults; 79 on ADHD MED 18–50 y	ADHD MED: 88.3% stimulants (52.2% dextroamphetamine; 18% lisdexamfetamine; 11.7% methylphenidate), 11.7% non-stimulants (atomoxetine, bupropion, and clonidine).	Adults receiving ADHD medication had approximately 6% lower bone mineral density at the skull and thoracic spine than controls. No consistent differences were observed at appendicular sites, and treatment duration was not significantly associated with BMD.

ADHD, attention-deficit/hyperactivity disorder; AE, United Arab Emirates; AgRP, agouti-related peptide; AMP, amphetamine; ATX, atomoxetine; AU, Australia; BH, Bahrain; BMD, bone mineral density; BMI, Body Mass Index; BR, Brazil; BT, behavioral therapy; CAC, case–control study; CDC, Centers for Disease Control; CG, control group; CN, China; CRO, cross-sectional study; DD, desire to drink; DE, Germany; DSM, Diagnostic and Statistical Manual of Mental Disorders; EAT-26, Eating Attitudes Test-26; EO/DD, emotional overeating/disordered eating; ER, extended release; ES, Spain; IN, Iran; IR, immediate release; IQR, interquartile range; kcal, kilocalories; KR, Korea; LDL, low-density lipoprotein; LDX, lisdexamfetamine; LT, longitudinal; MED, medication; MPH, methylphenidate; MPH-IR, immediate-release methylphenidate; MPX, dex-methylphenidate; NL, Netherlands; NUS, nutritional supplement; ODD, oppositional defiant disorder; OROS, osmotic-release oral system; PC1-3, Principal components (dietary PCs); RCT, randomized controlled trial; RDW, red-cell distribution width; RS = retrospective; SATG, stimulant and antipsychotic; SCFA, short-chain fatty acid; SD, standard deviation; SDS, standard deviation score; SE, Sweden; STG, stimulant only; TR, Turkey; US, United States of America; WHO, World Health Organization; y = year.

## Data Availability

The data analyzed during this study are available from the corresponding author upon request.

## Appendix A

**Table A1:** Search strategy for PubMed and Scopus Databases.

Keywords	PubMed	Scopus
ADHD	attention deficit hyperactivity disorder[Mesh] OR “attention deficit hyperactivity disorder”[tiab] OR ADHD[tiab]	“attention deficit hyperactivity disorder” OR ADHD
	AND	
DIET	diet*[tiab] OR nutrition[tiab] OR “dietary pattern*”[tiab] OR “dietary intake”[tiab] OR eating[tiab]	diet* OR nutrition* OR “dietary pattern*” OR “dietary intake” OR eating OR supplement*
	AND	
MEDICATION	methylphenidate[tiab] OR lisdexamfetamine[tiab] OR amphetamine*[tiab] OR atomoxetine[tiab] OR guanfacine[tiab] OR clonidine[tiab]	methylphenidate OR lisdexamfetamine OR amphetamine* OR atomoxetine OR guanfacine OR clonidine
